# Efficacy and safety of esketamine combined with propofol for curative endoscopic resection in colorectum: a prospective, randomized controlled trial

**DOI:** 10.1186/s12871-024-02475-z

**Published:** 2024-03-09

**Authors:** Yimei Ma, Jiali Wang, Yuying Yang, Minmin Yao

**Affiliations:** Department of Anesthesia, Zhongshan Hospital, Fudan University, Shanghai, 200032 People’s Republic of China

**Keywords:** Esketamine, Propofol, Sedation, Curative endoscopy

## Abstract

**Background:**

Curative endoscopic resection is widely used to treat colonic polyps and early stage cancers. The anesthetic strategy commonly involves the use of propofol combined with a small dose of opioids for sedation. Adverse respiratory or cardiovascular events such as hypotension often occur when attempting to achieve the necessary level of sedation. Several studies have suggested its advantages owing to the anesthetic, analgesic, and sympathomimetic properties of esketamine. However, there are no reports on curative colorectal endoscopic resection. We designed this randomized controlled trial to assess the efficacy and safety of esketamine combined with propofol for sedation in patients undergoing curative colorectal endoscopic resection.

**Methods:**

A total of 166 patients who underwent curative colorectal endoscopic resection were randomly assigned to groups A (propofol + fentanyl) or E (propofol + esketamine). Ideal sedation was assessed using the MOAA/S scale and was achieved using TCI-propofol with different doses of fentanyl and esketamine. The propofol consumption and vasoactive drug dosages were recorded. Sedation-related times, adverse events, and satisfaction were recorded.

**Results:**

Of the 160 patients, the total propofol consumption was significantly lower in group E (*n* = 81) (300 mg) than in group A (*n* = 79) (350 mg). Hypotension and bradycardia were significantly lower in Group E than in Group A. The groups showed no significant differences in other adverse events, induction time, recovery time, or patient or endoscopist satisfaction.

**Conclusion:**

Compared to fentanyl, esketamine helps decrease propofol consumption and increases cardiovascular stability during curative colorectal endoscopic resection in American Society of Anesthesiologists Class I–III patients without affecting anesthesia, patient and endoscopist satisfaction, or other adverse events.

**Trial registration:**

The study was retrospectively registered at the Chinese Clinical Trial Registry (www.chictr.org.cn; registration number: ChiCTR2300069014 on 03/03/2023).

## Introduction

The incidence of colon polyps and early-stage colon cancer remains high in China. Curative endoscopic resection is now the primary method for mini-trauma, with a short operation duration, shorter length of stay, and rapid recovery. The current commonly used sedation strategy involves combining propofol with small doses of opioids such as remifentanil, sufentanyl, or fentanyl [1, 2]. Unlike examination, the curative procedure requires a deeper level of sedation to ensure that the patient remains immobile, as any movement could significantly affect the surgical outcomes. Unlike gastroscopic resection, significant expiratory movement of the belly due to airway obstruction also influences the operation. In order to achieve a satisfactory degree of sedation, much propofol and opioids are required; however, adverse respiratory or cardiovascular events such as hypoxia and hypotension often occur while using these sedatives. Consequently, an optimized regimen of anesthetic agents is highly required for successful surgical procedures.

Propofol is widely used for anesthesia sedation; however, it is associated with a higher risk of cardiovascular and respiratory depression in a dose-dependent manner [3]. Fentanyl has frequently been included for its analgesic properties to alleviate pain when patients require deep sedation during painful procedures [4, 5]. This conventional regimen is widely used in patients undergoing curative endoscopic resection. However, this combination usually results in hemodynamic instability and respiratory depression, which are associated with adverse events in clinical settings. An ideal adjacent drug that can help reduce propofol dosage without causing side effects could be of great value in improving clinical safety, particularly in patients with advanced age or multiple comorbidities, which are important risk factors for adverse events associated with propofol.

Esketamine, the s-enantiomer of ketamine, has more potent analgesic and sedative effects, particularly favorable sympathomimetic properties, with fewer adverse reactions than racemic ketamine [6, 7]. It is now a popular medicine that is widely used in various areas [8], helps decrease propofol dosage, reduces the incidence of respiratory depression, stabilizes hemodynamics [9–11], and improves postoperative recovery quality [12–14]. Although studies have reported the usefulness of esketamine in ERCP and endoscopic examination [9, 15], studies examining its use with propofol in curative colorectal endoscopic resection are still lacking. Our study aims to fill the notable gap, exploring the efficacy and safety of the esketamine-propofol combination in this specific and challenging procedure.

We hypothesized that combining propofol and esketamine for sedation might help reduce propofol dosage and, therefore, increase clinical safety compared to the conventional strategy for these patients. Accordingly, we assessed the efficacy and safety of esketamine versus fentanyl as an adjunct to propofol for sedation in patients undergoing curative colorectal endoscopic resection.

## Methods

This single-center, prospective, randomized clinical trial was conducted from November 30, 2021, to September 15, 2022. Ethical approval (B2021-713) was obtained from the Ethics Committee on October 28, 2021. The trial was retrospectively registered at ClinicalTrials.gov (ChiCTR2300069014) and followed the Consolidated Standards of Reporting Trials (CONSORT) reporting guidelines. Written informed consent was obtained from all the participating patients.

This study included patients scheduled for curative endoscopic resection under general anesthesia without intubation, aged 18 years or older, classified as class I–III according to the American Society of Anesthesiologists (ASA), and who provided informed consent. Patients were excluded if they had a history of unregulated or malignant hypertension, significant ischemic heart disease, psychiatric disease, chronic pain, pregnancy, seizure disorders, increased intracranial pressure, drug abuse, or allergy to planned medication. Patients with changes in surgical or anesthetic procedures were excluded from the study.

Patients were randomly assigned in a 1:1 ratio to receive either propofol or esketamine (Group E) or propofol and fentanyl (Group A) sedation. Randomization was performed using computer-generated random numbers (IBM SPSS version 26.0). An independent researcher collected data. The researcher, endoscopist, endoscopy nurse, and patients were blinded to the treatment allocation to ensure impartiality.

### Endoscopic procedure and monitoring

All patients adhered to a fasting period of at least six hours prior to their scheduled surgery. Following successful intravenous access, patients were directed to assume a semi-prone position, and all patients received oxygen via an oxygen mask at a flow rate of 3–5 L/min. During the procedure, heart rate (HR), oxygen saturation (SpO_2_), electrocardiogram, respiratory rate, and exhaled carbon dioxide concentration were monitored continuously. Noninvasive blood pressure and sedation levels were assessed using the Modified Observer’s Alertness/Sedation Scale (MOAA/S) at 5-minute intervals.

### Sedative intervention

An experienced anesthesiologist sedated the patients with propofol (Fresenius Kabi Deutschland GmbH, Germany, H20060284) target-controlled infusion (TCI, Marsh Model) in both groups. Once the plasma concentration reached the desired target of 1.5 mg/ml, Group E received 0.15 mg/kg of esketamine (Jiangsu Hengrui Pharmaceuticals Co., Ltd. China, H20193336), and Group A received 1 µg/kg of fentanyl (Yichang Humanwell Pharmaceuticals Co., Ltd., China, H20003688). Subsequently, the target propofol TCI level increased to 2.5 mg/ml. The assessment of the patient’s sedation state was conducted once the effective concentration (Ce) reached 2.5 mg/ml, after which the endoscopic operation commenced. During the procedure, the sedation level was assessed using the Modified Observer Alertness/Sedation (MOAA/S) scale. We targeted sedation levels with a MOAA/S score of 2. The TCI was increased or decreased in steps of 0.5 mg/ml. If the MOAA/S score decreased to less than one or airway obstruction occurred, the TCI propofol dose was decreased. If the anesthesiologist noticed MOAA/S was above 2, propofol TCI was upregulated, and esketamine 0.05 mg/kg or fentanyl 0.5 µg/kg was added. The maximum dose was 0.5 mg/kg esketamine or 5 µg/kg fentanyl. Propofol was discontinued at the end of the surgery, and the dose was recorded.

Intraoperative vasoactive drugs (atropine, ephedrine, and phenylephrine) were administered to maintain the hemodynamic parameters within 20% of the baseline measurement. If patients experienced oxygen desaturation (SpO_2_ < 90%) during the operation, the jaw was lifted to alleviate airway obstruction and increase the oxygen flow rate to 6 L. If this measure was ineffective, the nasopharyngeal ventilation tract was used to address the obstruction. A pressure face mask was used if the SpO_2_ dropped rapidly. Endotracheal intubation was always kept as the last-resort option. All the events and methods were recorded. Ondansetron (4 mg) was administered to prevent postoperative nausea and vomiting.

After the procedure, patients were transferred to the post-anesthesia care unit (PACU). A modified Aldrete Score was used to assess recovery from anesthesia. Patients were considered ready for discharge when their Aldrete Score was 9 or higher, or equal to their preprocedural score, and without any significant adverse effects.

### Outcome assessment

The primary endpoint was the total propofol consumption in each group. The secondary endpoints were changes in vital parameters (mean arterial pressure, HR and respiratory rate), vasoactive drug dosage (including atropine, ephedrine, and phenylephrine), sedation-related time (effective and recovery time), patient and endoscopist satisfaction, and sedation-related adverse events.

Sedation levels were assessed using a MOAA/S. A MOAA/S score of 5 signifies that the patient remains alert and responds promptly to a name spoken in a normal tone, whereas a MOAA/S score of 0 indicates that the patient exhibits no response to noxious stimuli [16]. The effective time was defined as the time from drug administration to recovery until an MOAA/S score of 2 was achieved. The recovery time was defined as the time from drug withdrawal to the return of the MOAA/S score to 4. Patient and endoscopist satisfaction was measured using a 10-point visual analog scale (VAS, ranging from 0 to 10 ), with 0 representing extraordinarily unsatisfied and 10 representing excellent satisfaction.

Adverse events were defined according to the guidelines of the World SIVA International Sedation Task Force [17]. Respiratory events mainly refer to decreased oxygen saturation (SpO_2_ < 90%), classified as oxygen desaturation (SpO_2_, 75–90% for < 60 s), and severe oxygen desaturation (SpO_2_ < 75% at any time or prolonged SpO_2_ < 90% for > 60 s). Cardiovascular events included hypotension, hypertension, bradycardia, and tachycardia, which were defined as changes > 20% from the baseline value (the value before the procedure). Postoperative pain was assessed using a VAS ranging from 0 to 9. Postoperative nausea and vomiting (PONV) was assessed using a 5-point Likert scoring system and classified as none, mild, moderate, and severe [18]. Mood states were ranked from 0 to 100 in five categories: anxious to composed, hostile to agreeable, depressed to elated, tired to energetic, and confused to clear head. All subjective perceptions were assessed before PACU discharge and the next day at 7 a.m.

### Statistical analysis

Esketamine is expected to help reduce propofol dose by 15%. Based on a previous retrospective trial, the average propofol dose was 580 mg, with a standard deviation of 180 mg. With a statistical power of 80% and a two-tailed type-I error rate of 5%, 152 cases were needed to obtain statistically significant results. The sample size was increased to 166 to allow participant withdrawal and loss to follow-up.

All the analyses were based on the intention-to-treat principle. Variables are reported as numbers (percentages), means (SDs), or medians (IQRs), as appropriate. We used the Shapiro–Wilk test to assess whether continuous data were normally distributed. We performed a group comparison using the χ^2^ test or Fisher’s exact test for categorical variables and the two-tailed t-test or Mann–Whitney U test for continuous variables, when appropriate. Bonferroni correction was applied to account for multiple comparisons between groups. Statistical significance was set at *P* < .05. Statistical analyses were performed using IBM SPSS software (version 26.0; IBM Corp., Armonk, NY, USA).

## Results

### Patients

**Fig. 1:**
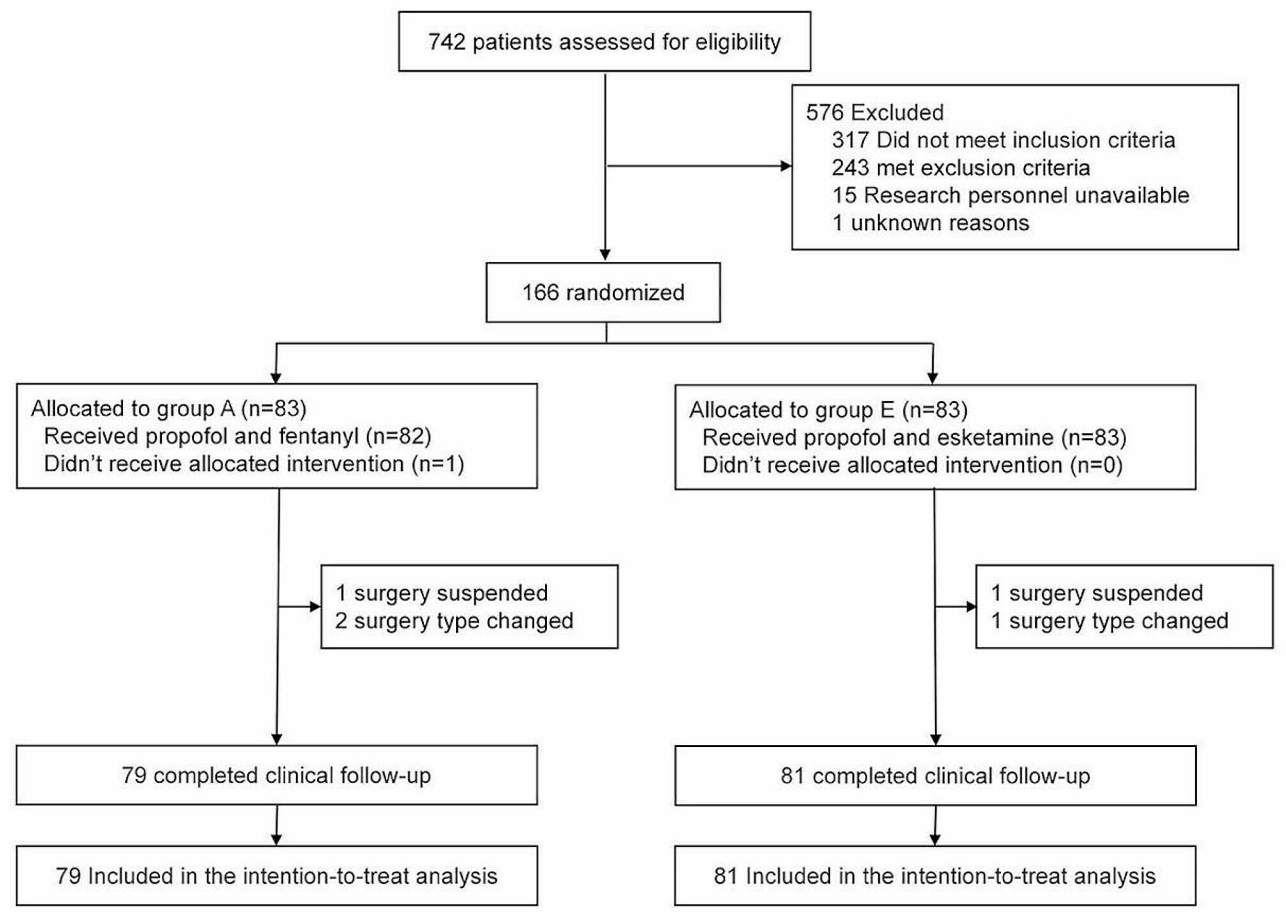
CONSORT flow diagram

We screened 792 patients, and 166 patients who underwent curative endoscopic resection of the colorectum were randomized into Groups A (propofol + fentanyl) and E (propofol + esketamine) (Fig. 1). Six patients withdrew from this study. The strategy was changed to general anesthesia for one patient, and the procedure was not executed according to the plan. Surgery was suspended in two patients because of poor bowel preparation. Three patients did not undergo endoscopic curative surgery because of the high possibility of aggressive tumors but only underwent examination and labeling. Ultimately, 160 patients were included in the final analysis (79 and 81 patients in Groups A and E, respectively).

There were no significant differences in the demographic and clinical characteristics at baseline, including age, BMI, comorbidities, and Mallampati score (*P* > .05) (Table 1).

**Table 1:** Baseline demographic and clinical characteristic

Characteristic	Fentanyl (*n* = 79)	Esketamine (*n* = 81)	p
Age, y	60.5 ± 10.8	61.0 ± 11.1	0.782
Sex, n%			0.973
Male	48 (60.8)	49 (60.5)	
Female	31 (39.2)	17 (39.5)	
Height, cm	166.2 ± 7.4	166.3 ± 6.8	0.934
Weight, kg	64.2 ± 11.2	66.3 ± 10.9	0.221
BMI, kg/m^2^	23.2 ± 3.5	23.9 ± 3.1	0.169
Smoker	7 (8.9)	8 (9.9)	0.826
Alcohol	2 (2.5)	3 (3.7)	0.67
Comorbidities			
Hypertension	20 (25.3)	27 (33.3)	0.266
Diabetes	6 (7.6)	13 (16)	0.098
Cerebrovascular disease	4 (5.1)	6 (7.4)	0.746
Chronic pulmonary disease	1 (1.3)	2 (2.5)	1
ASA			0.229
I	22 (27.8)	16 (19.8)	
II	57 (72.2)	65 (80.2)	
Mallampati			0.437
I	5 (6.3)	4 (4.9)	
II	64 (81.0)	63 (77.8)	
III	10 (12.7)	14 (17.3)	
Baseline measurement			
SpO2	99.3 ± 0.9	99.3 ± 1.2	0.902
Heart rate	73.9 ± 10.8	73.6 ± 11.3	0.867
SBP	134.8 ± 15.3	132.7 ± 17.6	0.442
DBP	78.7 ± 11.1	73.9 ± 10.8	0.208
Surgery type			0.13
EMR	53 (67.1)	63 (77.8)	
ESD	26 (32.9)	18 (22.2)	

**Abbreviations**: BMI, body mass index (calculated as weight in kilograms divided by height in meters squared); ASA, American Society of Anesthesiologists; SBP, systolic blood pressure; DBP, diastolic blood pressure; EMR, endoscopic mucosal resection; ESD, endoscopic submucosal dissection

Data are presented as the mean ± SD unless otherwise indicated, with the P-value representing the significance level for differences between groups A and E (Student’s t-test or Pearson’s χ^2^ test)

### Primary outcome

The propofol dose is an important index for evaluating its safety and effectiveness. As shown in Table 2, the total propofol dose was significantly lower in Group E (300 [235–395] mg) than in Group A (350 [290–420] mg) (*P* = .009). After adjusting for the body weight and operation time, the average propofol consumption was still much less in Group E (9.5 ± 3.1 vs. 10.7 ± 3.1 mg/kg/h) (*P* = .022).

**Table 2:** Surgical procedure and intraoperative parameters

Variable	Fentanyl (*n* = 79)	Esketamine (*n* = 81)	p
Total propofol dose, mg	350 (290–420)	300 (235–395)	0.009
Average propofol consumption, mg/kg/h	10.7 ± 3.1	9.5 ± 3.1	0.022
Induction time, min	2 (2–3)	2 (2–3)	0.215
Operation time, min	34 (24–46)	31 (21–43.5)	0.474
Awake time, min	7 (5–10)	9 (6–12)	0.087
Recovery time, min	30 (24–34)	31 (23.5–33.5)	0.882
Patient Satisfaction	5	5	1
Endoscopist satisfaction	5	5	1
Surgery difficulty			0.201
1	59 (74.7)	67 (82.7)	
2	18 (22.8)	14 (17.3)	
3	2 (2.5)	0 (0)	

Data are presented as mean ± SD, n (%), or median (IQRs), with the P-value representing the significance level for differences between groups A and E (Student’s t-test, Mann–Whitney U test, Pearson’s χ^2^ test, or Fisher exact test)

### Secondary outcomes

Overall, there was no significant difference in surgical difficulty (*P* = .201). The operation times of the two groups were comparable (E vs. A: 31 [21–43.5] vs. 34 [24–46] min) (*P* = .474). This strategy did not significantly influence the induction and recovery times, and all patients and endoscopists were satisfied with the sedation regimen (Table 2).

Under similar procedural conditions, patients in Group E showed a lower incidence of hypotension (E vs. A:27/81 [33.3%] vs. 52/79 [65.8%]) (*P* = .001). None of the patients in either group had hypertension. The incidence of bradycardia was significantly lower in Group E than in Group A (E vs. A:0/81 [0%] vs. 11/79 [13.9%]) (*P* = .001). Only one patient experienced tachycardia. The results showed that patients in Group E received fewer vasoactive drugs, and the need for ephedrine was significantly lower (E vs. A:9/81 [11.1%] vs. 26/79 [32.9%]) (*P* = .001) (Table 3). However, no significant differences were observed after phenylephrine treatment (Table 3).

The incidence of oxygen desaturation was similar between groups (E vs. A:12/81 [14.8%] vs. 20/79 [25.3%]) (*P* = .097). Respiratory adverse events were observed and resolved over time. There was a single instance of severe oxygen desaturation, during which we halted the propofol infusion, performed a jaw lift, and inserted a nasopharyngeal ventilation tract to alleviate airway obstruction while increasing the oxygen flow rate to 6 L. This issue was successfully resolved with face mask ventilation within a few minutes (Table 3).

There were no significant intergroup differences in any of the other postoperative adverse events. Two (2.5%) patients in Group E and one (1.3%) patient in Group A experienced postoperative pain. Only one patient experienced PONV after recovery, which was alleviated within an hour. In addition, mood states were assessed before PACU discharge and on postoperative day 1 at 7 a.m., and there was no significant difference in psychotic symptoms, such as nightmares or hallucinations, until postoperative day 1. The detailed assessment method is described in the Outcome Assessment section of the Methods section (Table 3).

**Table 3:** Sedation-related adverse events

Events	Fentanyl (*n* = 79)	Esketamine (*n* = 81)	p
Oxygen desaturation	20 (25.3)	12 (14.8)	0.097
Severe oxygen desaturation	1 (1.3)	0 (0)	0.494
Hypotension	52 (65.8)	27 (33.3)	0.001
Hypertension	0 (0)	0 (0)	
Bradycardia	11 (13.9)	0 (0)	0.001
Tachycardia	1 (1.3)	0 (0)	0.494
Need for ephedrine	26 (32.9)	9 (11.1)	0.001
Need for phenylephrine	28 (35.4)	19 (23.5)	0.096
PONV	0 (0)	1 (1.2)	0.506
Pain	1 (1.3)	2 (2.5)	0.497
Bad mood state	1 (1.3)	1 (1.2)	0.745

**Abbreviations**: PONV, postoperative nausea and vomiting

Data are presented as mean ± SD, n (%), or median (IQRs), with the P-value representing the significance level for differences between groups A and E (Student’s t-test, Mann–Whitney U test, Pearson’s χ^2^ test, or Fisher exact test)

## Discussion

To our knowledge, this is the first clinical trial to evaluate the efficacy and safety of esketamine combined with propofol in the field of curative endoscopic resection of the colorectum. Our randomized controlled trial showed that esketamine decreased propofol requirement compared to propofol combined with fentanyl in patients undergoing curative endoscopic resection of the colorectum. Under identical sedation conditions, esketamine was found to contribute to a more stabilized cardiovascular system. However, it did not lead to any enhancement in respiration and had no discernible impact on the induction and recovery times or the occurrence of other adverse effects. All the patients and endoscopists were satisfied with the procedure.

During therapeutic colorectal endoscopy, insufflation is a crucial procedure that heavily impacts hemodynamics and respiratory function through increased intra-abdominal pressure. Multiple and some difficult-to-access polyps, especially, often lead to significantly extended surgical times, which result in increased requirement of propofol consumption and intensify the challenges in stable physiological states. So, there is a specific higher demand for anesthesia in therapeutic colorectal endoscopy to endoscopy examination or ERCP to ensure smooth surgery progression and the safety of the patient.

Esketamine is an N-methyl-D-aspartate receptor antagonist and an anesthetic agent with analgesic effects and sympathomimetic properties [8]. Esketamine can counteract the cardiopulmonary depressive effects of propofol, and numerous studies have demonstrated that the combination of propofol and esketamine reduces the required propofol dosage compared to propofol combined with fentanyl during painless sedative surgery [9, 19]. Both these factors support esketamine as an ideal adjacent medicine.

There was a correlation between these effects and dosage. The propofol dosage was determined according to previous studies [20, 21]. In our trial, the propofol TCI model was used to achieve more stable hemodynamics and reduce respiratory depression [22]. Evidence suggests that combining 0.1–0.2 mg/kg of esketamine with propofol is both effective and safe for painless gastrointestinal endoscopy [11]. Eberl et al. [9] showed that a dose of 0.15 mg/kg of esketamine was used for endoscopic retrograde cholangiopancreatography as per their requirement. Given the absence of a universally agreed-upon optimal dose of esketamine supplementation, our protocol utilizes 0.15 mg/kg esketamine in conjunction with propofol for sedation, taking into account both clinical efficacy and safety.

Our results showed that 0.15 mg/kg esketamine reduced total propofol dosage by 14.3% compared to fentanyl while providing equal quality of sedation. In light of varying patient weights and surgical duration, the standardized dose of propofol also showed an average of 1.2 mg/kg/h less. Since propofol dosage is an important index for safety evaluation, the results indicated that 0.15 mg/kg esketamine provided a better quality sedation regimen than fentanyl when combined with propofol TCI of 2.5 mg/kg.

Opioids like fentanyl are known to cause histamine release, leading to decreased systemic vascular resistance and blood pressure [23]. In our study, patients in the esketamine group exhibited a lower incidence of hypotension than those in the fentanyl group did. This was in accordance with previous studies that esketamine leads to a more stable hemodynamic status than opioids when combined with propofol [9, 10, 24]. Apart from the low propofol dosage, a more stable hemodynamic status may also be attributed to the sympathomimetic properties of low-dose esketamine, which counteracts propofol-induced circulatory depression. A recent study focused on the effects of esketamine on hypotension and desaturation in bidirectional endoscopy also supported our result [15].

The incidence of bradycardia was lower in the esketamine group than in the fentanyl group; however, there was no increase in the incidence of tachycardia in the present study. This study found a higher incidence of tachycardia in the esketamine group [24]. It is possible that the higher propofol dose used in this study contributed to this outcome. Second, our control group was administered fentanyl, which usually has a higher probability of bradycardia owing to its cardiovascular depression properties [23].

This study revealed no statistically significant differences in operation, induction, or recovery times between the two groups. This is consistent with previous studies showing that low-dose esketamine did not affect recovery time compared with opioids as an adjunct to propofol [25]. Another study found that combining propofol with esketamine significantly shortened the recovery time in elderly patients [26], which could be attributed to a reduction in propofol requirement in these patients.

Several trials [10, 25] have shown that combining esketamine with propofol sedation improves respiratory function. Song et al. reported that low-dose esketamine resulted in an approximately 61% reduction in the incidence of desaturation and hypotension in endoscopic examination [15]. However, in our current trial, despite a decreased dosage of propofol and the respiratory stimulatory properties of low-dose esketamine [27], there was no change in respiratory events. This could be attributed to the fact that the majority of patients had a Mallampati score ≤ 2, and the reduction in dosage may not have been sufficient to decrease the incidence of such events. Moreover, the propofol TCI model reduces the incidence of respiratory depression [22]. Undesirable movements, which involve significant diaphragmatic movement owing to airway obstruction, can potentially result in severe injuries during surgery. Therefore, airway obstruction usually resolves before desaturation. Liu’s [24] and Eberl’s [9] study also showed that the combination of propofol with esketamine (at doses between 0.15 mg/kg and 0.5 mg/kg) did not affect the incidence of hypoxemia compared to opioids.

No significant differences were noted in the risk of psychotomimetic effects, possibly because, in our clinical trial, TCI with propofol was initiated before the administration of esketamine. This initiation of TCI inhibits ketamine-induced c-fos expression in the posterior cingulate cortex through GABA receptor activation [28].

Esketamine has been widely used in many clinical settings, such as day surgery cases and is one of the core elements of non-opioid regimen and multi-disciplinary analgesia. This study has proved that esketamine combined with propofol significantly decreased the total and standardized dose of propofol compared with fentanyl and maintained more stable hemodynamics. Also, a larger sample size might help reveal more benefits of applying esketamine in therapeutic endoscopy of the colorectum, especially in respiratory outcomes.

Our study had several limitations. First, this was a small single-center study, which might affect the generalizability of our findings. Thus, a multicenter study is required to confirm our results. Second, a previous study determined that the dose of esketamine may not have an analgesic effect on fentanyl. Nevertheless, there is a lack of data on the equianalgesic doses of these two drugs. To account for this disparity, we used the same sedation targets in our study. Third, the duration of surgery and the extent of injury may be influenced by the various surgeons involved, potentially affecting the required propofol dosage. Finally, the sample size may not have been sufficiently large to evaluate the effect of hypoxia or the need for ventilation, which is a relatively small probability. A larger sample size and proper patients with a high Mallampti score or older age will be required to explore respiratory effects in future studies.

## Conclusions

Compared with the traditional sedation strategy of propofol TCI combined with fentanyl, propofol TCI combined with esketamine reduced propofol dosage and increased cardiovascular stability for curative endoscopic resection in the colorectum in ASA class I–III patients without affecting the anesthetic procedure, patient and endoscopist satisfaction, or other adverse events.

## Data Availability

All data generated or analyzed during this study were included in the published article. These data will be available from the corresponding author on reasonable request.

## Abbreviations

ASA: American Society of Anesthesiologists
MOAA/S: Modified Observer’s Alertness/Sedation Scale
PACU: Post Anesthesia Care Unit
HR: heart rate
SPO_2_: oxygen saturation
TCI: target-controlled infusion
Ce: effective concentration
VAS: visual analog scale
PONV: postoperative nausea and vomiting
BMI: Body mass index
SBP: systolic blood pressure
DBP: diastolic blood pressure
EMR: endoscopic mucosal resection
ESD: endoscopic submucosal dissection
